# DICOM datasets for reproducible neuroimaging research across manufacturers and software versions

**DOI:** 10.1038/s41597-025-05503-w

**Published:** 2025-07-09

**Authors:** Christopher Rorden, Benoît Béranger, Hu Cheng, Matthew Clemence, Clément Debacker, Brice Fernandez, Yaroslav O. Halchenko, Michael P. Harms, Bharath Holla, Isaiah Innis, Joost P. A. Kuijer, Daniel Levitas, Krisanne Litinas, Jeffrey Luci, Roger Newman-Norlund, Scott Peltier, Wolfgang Rehwald, Robert I. Reid, Baxter Rogers, Christopher G. Schwarz, Jaemin Shin, Venkatasubramanian Ganesan, Sandeep Ganji, Paul S. Morgan

**Affiliations:** 1 McCausland Center for Brain Imaging, Department of Psychology, University of South Carolina, Columbia, SC 29208 USA; 2 CENIR, Paris Brain Institute - ICM, Hôpital Pitié-Salpêtrière de Sorbonne Université, Paris, France; 3 Department of Psychological and Brain Sciences, Indiana University, Bloomington, IN 47405 USA; 4 Philips Healthcare, Farnborough, UK; 5 Université Paris Cité, Institute of Psychiatry and Neuroscience of Paris (IPNP), INSERM U1266, IMA-Brain team, 75014 Paris, France; 6 GE HealthCare, Buc, France; 7 Department of Psychological and Brain Sciences, Department of Computer Science, Hanover, NH USA; 8 Department of Psychiatry, Washington University School of Medicine, St. Louis, MO USA; 9 Department of Psychiatry, Integrative Medicine, National Institute of Mental Health and Neuro-Sciences (NIMHANS), Bengaluru, India; 10 Radiology & Nuclear Medicine Amsterdam UMC, Amsterdam, The Netherlands; 11 Functional MRI Laboratory, Department of Radiology, University of Michigan, Ann Arbor, MI USA; 12 Center for Advanced Human Brain Imaging Research, Rutgers University, Piscataway, NJ USA; 13 Department of Psychiatry, Rutgers Robert Wood Johnson Medical School, Piscataway, NJ USA; 14 Siemens Medical Solutions USA, Inc., Malvern, PA USA; 15 Department of Radiology, Mayo Clinic, Rochester, MN 56001 USA; 16 Vanderbilt University Institute of Imaging Science, Nashville, TN 37232 USA; 17 Vanderbilt University Medical Center Department of Radiology and Radiological Sciences, Nashville, TN 37232 USA; 18 Vanderbilt University Department of Biomedical Engineering, Nashville, TN 37232 USA; 19 Vanderbilt University Medical Center Department of Psychiatry and Behavioral Sciences, Nashville, TN 37232 USA; 20 GE Healthcare, GE Healthcare, New York, NY USA; 21 Philips, Cambridge, MA 02142 USA; 22 School of Medicine, University of Nottingham, Nottingham, UK; 23 NIHR Nottingham Biomedical Research Centre, Nottingham, UK

**Keywords:** Neuroscience, Medical research, Neurology

## Abstract

DICOM is an industry-standard for medical imaging data targeted at interoperability across systems. This enables transfer, storage and processing of imaging data regardless of the manufacturer. Pragmatically, manufacturers often store detailed acquisition parameters in private rather than public DICOM tags. In parallel, the DICOM standard itself has gradually evolved by introducing new public tags and properties to better capture emerging imaging technologies. Accurately extracting these details is essential for reproducible neuroimaging research. To address this need, we created a series of DICOM datasets illustrating how various manufacturers encode acquisition details that are critical for modern processing and analysis. These minimal test cases, covering CT and MR modalities, highlight manufacturer-specific conventions, including the use of public tags, private tags, and proprietary data structures. For each DICOM dataset, we provide corresponding NIfTI-formatted images with metadata JSON files following the BIDS standard, using consistent terminology to mitigate variations in how manufacturers encode acquisition details. Our repository provides validation datasets for any tool that is intended to extract acquisition details from medical imaging data.

## Background & Summary

Reproducibility is a critical challenge in neuroimaging research^1^. Most analyses involve multiple stages of image processing and complex statistical modeling to mitigate noise and identify meaningful signals^2^. These processes require precise knowledge of acquisition parameters, such as slice timing and phase encoding polarity. Studies aiming to aggregate data across sites must also address variability between scanners in order to ensure generalizability^3^. Consequently, neuroimaging researchers must be able to reliably extract details about the acquisition parameters. This task is facilitated by the Digital Imaging and Communications in Medicine (DICOM) standard^4^, which dominates medical imaging, promoting interoperability across tools and manufacturers. However, the rapid evolution of imaging technologies often outpaces consensus-based updates to the standard, leading manufacturers to use self-defined (“private”) metadata tags, which are, ideally, later integrated into the DICOM definition as standardized (“public”) tags. We provide a comprehensive collection of DICOM images spanning various manufacturers, modalities, and software versions to address this challenge. We also offer ground truth values for the imaging parameters that are crucial for reproducibility. These datasets enable tool developers to ensure robust and reproducible analyses of neuroimaging data.

Historically, each neuroimaging team used its own idiosyncratic method to provide sequence details for analysis with neuroimaging pipelines. The Brain Imaging Data Structure (BIDS)^5,6^ provided a more standardized framework for organizing and describing imaging datasets, defining the imaging format (voxel intensity stored in NIfTI), imaging parameters (in human-readable JSON text files using manufacturer agnostic terminology), and file naming (providing hints for intention), as well as the relevant non-imaging details of an experiment (e.g., participant behavioral data and demographics). The BIDS format allows for automated analyses of datasets regardless of scale, aids reproducibility, and facilitates data sharing and reuse. While this intentionally constrained format is considerably simpler than DICOM, it is worth noting that since the source data has DICOM format, adopting the BIDS structure does not replace the arduous^7^ task of accurately extracting acquisition details from raw imaging data. Therefore, repositories that share neuroimaging data in BIDS format^8^ require data providers to extract acquisition details prior to sharing, while DICOM repositories^9,10^ require data users to extract imaging parameters prior to analysis. These needs have become increasingly acute as public funding agencies expect scientists to share large datasets and clinical teams are aggregating huge datasets to empower precision medicine. We aim to support both approaches, providing validation datasets that ensure that imaging data and metadata can be determined from DICOM data regardless of scanner manufacturer and software version. While our team has focused on generating domain-specific BIDS/NIfTI formats, the resulting validation repositories also support efforts to extract standardized parameters across manufacturers for teams that choose to retain the DICOM format^11^.

Prior work in this topic includes the seminal “Rosetta bit” project^12^ which highlighted the importance of validation datasets in neuroimaging and providing gold-standard conversions of DICOM to NIfTI images. However, that project predated BIDS, and therefore while it provided a validation for voxel intensities and spatial properties, it did not provide sequence details crucial for image processing within a site and data harmonization across sites. Likewise, Rutherford and colleagues^13^ synthesized DICOM datasets to evaluate the performance of de-identification algorithms, but did not address acquisition details. Our datasets extend these traditions, providing updated resources to support modern interpretations of the DICOM standard in general, as well as the introduction of the enhanced DICOM format^14^.

Therefore, our overarching objective is to provide validation DICOM datasets that demonstrate how different manufacturers and different software versions store acquisition parameters. Our datasets include both the original DICOM datasets as well as the known solutions for critical acquisition parameters (using text files in the BIDS specification). Our datasets provide minimal test cases for understanding manufacturer-specific conventions, including private DICOM tags, and demonstrate edge cases that require careful interpretation. These repositories aid in developing and maintaining tools that read DICOM images like dcm2niix, dicm2nii and SPM^15^. By providing BIDS-compatible NIfTI images alongside standardized metadata, we offer a practical resource for improving data conversion and enhancing reproducibility.

## Methods

We have assembled a collection of 36 distinct DICOM modules publicly available on Zenodo^16^ (Table 1; with mirrors on GitHub) designed to illustrate the diversity of images observed in the neuroimaging domain. Where possible, the datasets use low-resolution images and relatively few volumes with the aim of providing concise examples of specific use cases. This is in contrast to traditional research repositories where high spatial resolution and many observations are considered beneficial. We have curated our examples into specific repositories highlighting specific challenges. The rationale for each of these repositories is included in its “README.md” text file. A brief overview of the DICOM and BIDS methods for storing sequence information will provide context for the challenge of interpreting these validation datasets.

**Table 1:** DICOM modules with validated conversion to a harmonized terminology defined by the BIDS specification.

Repository Name	Manufacturer	Modality	Comments
dcm_qa	S	fmri	Image orientation, total readout time, multi-band
dcm_qa_12bit	S	fmri	12-bit signed and unsigned voxel intensities
dcm_qa_asl	S	asl	Arterial spin labeling
dcm_qa_canon	C	dwi	Canon 6.0 classic DICOM images
dcm_qa_canon_61	C	dwi	Canon 6.1 images saved as enhanced and classic DICOM
dcm_qa_canon_enh	C	dwi	Canon 6.0 enhanced DICOM images
dcm_qa_cs_dl	G,P,S	smri	Compressed Sensing (CS) and the Deep Learning filters
dcm_qa_ct	G,P	ct	Computerized Axial Tomography
dcm_qa_decubitus	S	mri	Head first decubitus images
dcm_qa_decubitus_ge	G	smri	Head first decubitus image
dcm_qa_deident	S	smri	De-identification method parameters
dcm_qa_dti	S	dwi	Diffusion directions with various slice angulations
dcm_qa_enh	C,P,S	mri	Enhanced DICOM
dcm_qa_fmap	G,S	fmap	Field mapping
dcm_qa_ge	G	mri	Slice timing, acquisition acceleration
dcm_qa_me	S	mri	Multi-echo sequences
dcm_qa_mosaic	S	fmri	Mosaic images with reverse image numbering
dcm_qa_mprage	S	smri	Magnetization Prepared - RApid Gradient Echo sequences
dcm_qa_nih	G,S	fmri	Phase encoding polarity
dcm_qa_pdt2	S	mri	Proton-Density and T2-weighted imaging
dcm_qa_philips	P	dwi	Philips classic and enhanced DICOMs
dcm_qa_philips_asl	P	asl	Philips classic DICOM arterial spin labeling
dcm_qa_philips_asl_enh	P	asl	Philips enhanced DICOM arterial spin labeling
dcm_qa_philips_dwi	P	dwi	Philips diffusion with unusual volume ordering
dcm_qa_philips_enh	P	mri	Philips enhanced DICOM
dcm_qa_polar	G	fmap	GE reversed phase encoding polarity
dcm_qa_sag	S	fmri	Sagittal diffusion images
dcm_qa_stc	G,S,U	fmri	Slice timing correction
dcm_qa_table	G,P,S	fmri	Tracking table position
dcm_qa_toshiba	C	dwi	Toshiba 5.0 classic DICOMs
dcm_qa_trt	G	fmri	Total Readout Time
dcm_qa_ts	S	fmri	Various DICOM transfer syntaxes
dcm_qa_uih	U	mri	United Imaging Healthcare (“UIH”) MRI scanners
dcm_qa_xa30	S	mri	Siemens XA30 enhanced DICOMs
dcm_qa_xa30i	S	mri	Siemens XA30 classic (interoperability) DICOM
dcm_qa_xa60	S	mri	Siemens XA60/XA61 enhanced DICOMs

The Manufacturer refers to the vendor for hardware (C = Canon/Toshiba, G = General Electric, P = Philips, S = Siemens, U = United Imaging Healthcare). The Comments describe edge cases exhibited by the dataset. Modality refers to type of image (asl = arterial spin labeling, dwi = diffusion weighted imaging, fmap = fieldmap, fmri = functional, mri = dataset with multiple modalities, smri = structural, ct = computerized axial tomography).

While DICOM files can contain many classes of data (e.g. sounds, waveforms, text documents)^4^, here we focus on files that contain images. A DICOM image file contains both the image data (voxel intensity values) as well as a series of tags that describe the image acquisition. Each DICOM tag is defined as two 16-bit hexadecimal numbers referred to as “group” and “element”, commonly written as text in the form <gggg,eeee>. All even numbered groups refer to public tags that are defined by the DICOM specification. Some public tags are required for a specific image modality (for example, the numeric <0018,0081> “Echo Time” is required for MR images) while others are optional (e.g., the string <0018,1020> “Software Versions” is optional). In contrast, odd numbered groups are private tags that the manufacturer can define. In the same way that typed programming languages define variables as strings, integers or floating point numbers, each DICOM tag is associated with a ‘Value Representation’ (VR) that defines the type of the data. While most DICOM tags store variables of a fixed type (e.g., a string, or an array of integers), some manufacturers use the VR ‘Other Byte’ (OB) to store sequence details using their own proprietary formats. Classic DICOM objects typically only store a single 2D slice in each file. For 4D time series such as functional MRI and diffusion imaging, this can result in tens of thousands of files for a single imaging series. Some manufacturers (Siemens and United Imaging Healthcare) provide the option to save all slices from a 3D volume as tiles (‘mosaics’) in a single DICOM file, dramatically reducing the number of files per series (at the cost of non-compliance with the DICOM standard and requiring tools to de-interlace these 2D mosaics into a 3D volume). More recently, the ‘enhanced DICOM’ specification^14^ allows saving multi-frame data where entire 3D and 4D series are stored in a single DICOM file. While complex, the DICOM standard provides tremendous flexibility and can work across a broad range of medical images.

The BIDS specification stores computed tomography (CT) and magnetic resonance imaging (MRI) data via two files. The pixel data and spatial properties (e.g., slice angulation) are saved as a binary NIfTI format file (which itself contains its own limited metadata) while other acquisition meta data are stored in a human-readable text file in the JSON format of key:value pairs. For example, the numeric value ‘*“EchoTime”: 0.03*’ and the string value ‘*“SoftwareVersions”: “syngo MR B17”*’. BIDS stores 3D anatomical images as a single file, and most 4D functional and diffusion time-series as a single file (though note, for multi-echo time series, each echo is stored as a separate file). This format has a more limited scope and is more constrained than DICOM. For example, all slices in a 3D NIfTI volume must be equidistant, while the DICOM format allows for variable slice distances. Another example is that DICOM supports many formats for compressing the voxel data (referred to as transfer syntaxes), with many of these compression schemes essentially unique for medical imaging (e.g., lossless JPEG formats with 16-bit precision were not widely adopted outside DICOM). In contrast, BIDS images can either be compressed or use the old but ubiquitous gzip file-level compression. The human-readable nature of BIDS JSONs requires floating-point numbers to be stored as ASCII text, which can introduce rounding errors and differences in precision compared to binary representations. This limitation is particularly relevant for our validation datasets, as tools that use different conventions for storing values may detect minor discrepancies between their outputs and the provided reference values. As a result, a degree of tolerance may be necessary to distinguish meaningful errors from negligible differences in value representation.

### Common terminology across manufacturers

Many terms are clearly defined by the DICOM standard and are unambiguous across manufacturers. Since both DICOM and the BIDS specification attempt to describe the parameters used to acquire imaging data, it is unsurprising that many DICOM public tags map directly to BIDS keys (Table 2). However, it is worth noting that different manufacturers interpret some public DICOM tags differently. For example, some MR sequences have both brief and long repetition times, and some manufacturers report the brief duration while others use the long duration for the public tag <0018, 0080> “Repetition Time”. In contrast, the BIDS standard attempts to disambiguate different intervals, hence the BIDS keys “RepetitionTime”, “RepetitionTimeExcitation” and “RepetitionTimePreparation”.

**Table 2:** Some DICOM tags have a one-to-one correspondence with BIDS fields.

BIDS Field	DICOM Tag
BodyPart	<0018,0015>
EchoTime	<0018,0081>
FlipAngle	<0018,1314>
InstitutionAddress	<0008,0081>
InstitutionalDepartmentName	<0008,1040>
InstitutionName	<0008,0080>
InversionTime	<0018,0082>
MagneticFieldStrength	<0018,0087>
Manufacturer	<0008,0070>
ManufacturersModelName	<0008,1090>
MRAcquisitionType	<0018,0023>
MTState	<0018,9020>
ParallelAcquisitionTechnique	<0018,9078>
ParallelReductionFactorInPlane	<0018,9069>
ParallelReductionFactorOutOfPlane	<0018,9155>
PartialFourierDirection	<0018,9036>
ReceiveCoilName	<0018,1250>
RepetitionTime	<0018,0080>*
RepetitionTimeExcitation	<0018, 0080>*
ScanningSequence	<0018,0020>
ScanOptions	<0018,0022>
SequenceName	<0018,0024>
SequenceVariant	<0018,0021>
SoftwareVersions	<0018,1020>
SpoilingState	<0018,0021>
StationName	<0008,1010>

In some cases, classic DICOM uses a different tag than enhanced DICOM.

### Manufacturer specific terminology and missing data

Some BIDS keys map onto private tags used by specific manufacturers (Table 3). The table presents private DICOM tags that map directly onto a single BIDS key:value pair. Manufacturers can also record information in public DICOM tags that are embedded inside private tags, with values that conflict with the public tag information stored at the root level (e.g., our ‘dcm_qa_philips’ repository demonstrates this with the public tag <0020,0032> “Image Position (Patient)”). In addition, as noted previously, some manufacturers use OB data chunks to encode variables using their own proprietary data structures. Furthermore, different manufacturers use different conventions to encode the spatial direction for the diffusion-sensitizing directions/vectors that accompany diffusion imaging (with some using world space and others using image space). Finally, the DICOM files generated by some manufacturers omit acquisition details that are needed by some BIDS-compliant pipelines. For example, Philips DICOM files do not provide the details required to populate the BIDS ‘SliceTiming’ array. This means that a user must either populate that information manually (for example, using ezBIDS^17^) or skip the slice time correction processing step that can improve statistical power in certain acquisition regimes^18^. Likewise, Philips DICOM files do not record the phase encoding *polarity* or readout-time parameters necessary for correcting spatial distortions in echo-planar acquisitions^19^. In addition to addressing variations in reporting data, validation datasets play a crucial role in identifying missing variables and errors in DICOM files. When data are absent, tools such as PET2BIDS^20^ can supplement missing information to enable subsequent analyses. For both, this enables manufacturers to correct these issues and allows tools to flag problematic data. A notable example is the recent Siemens enhanced data, where features like slice timing and the MultibandAccelerationFactor were incorrectly specified, as documented in our dcm_xa_61 repository. Given that both BIDS and DICOM are evolving community standards, a comprehensive listing is beyond the scope of this work. Instead, we provide hyperlinks to the formal specifications and refer readers to the respective websites for Tables 2, 3, which are best suited to track ongoing updates.

**Table 3:** Some manufacturer private DICOM tags have one-to-one translations to BIDS terminology.

BIDS Field	DICOM Tag
DwellTime	<0019,1018>
LabelingDuration	<0043,10A5>
MultibandAccelerationFactor	<0043,1083>
MultibandAccelerationFactor	<0021,1009>
ParallelReductionFactorInPlane	<0043,1083>
ParallelReductionFactorInPlane	<0021,1009>
ScanningSequence	<0021,105a>
SpoilingState	<0021,105B>
TablePosition	<0043,10B2>
TablePosition	<2005,143 C>
TablePosition	<0019,107 F>
TablePosition	<0019,1014>
TablePosition	<0021,1005>

This table provides private tags for General Electric (groups 0027 and 0043), Philips (groups 2001 and 2005), and Siemens (groups 0021, 0019, and 0051).

While the public DICOM tags and defined BIDS fields aim to standardize common acquisition parameters, in practice, manufacturers may use proprietary values or settings that lack an unambiguous one-to-one mapping. Our diverse datasets are intended to illustrate both standardization and areas of divergence. For example, the ‘dcm_qa_cs_dl’ repository includes MRI series from Canon, Philips, and Siemens where deep learning–based image reconstruction is employed, with vendor-specific settings such as different sharpening levels (e.g., off, low, or high). Detecting these proprietary settings can help identify inter-site differences and support statistical modeling when acquisition parameters vary. Thus, while our primary goal is to provide examples of commonalities across manufacturers, the datasets also capture important instances of manufacturer-specific variability.

### Third-party modifications

Additionally, some third-party DICOM PACS (Picture Archiving and Communication System) systems can modify each DICOM file they touch. This can introduce further challenges including renumbering of private tags, appending their own tags, or altering the compression scheme used for the voxel intensities (the transfer syntax). These modifications can make it more difficult to interpret the original manufacturer’s proprietary tags and may require additional troubleshooting or adjustments during the conversion process. Ensuring compatibility with such systems is an ongoing effort that underscores the importance of robust, adaptable conversion tools and detailed documentation. The ‘dcm_qa_ts’ repositories provide concrete examples of these situations.

## Data Records

All datasets are available from the dcm_validate master repository, which links to each validation dataset as a Git submodule. The repository is archived at Zenodo (10.5281/zenodo.15310934)^16^. Our validation dataset has a hierarchical structure, with specific edge cases (manufacturer, software, modality) illustrated in independent modular repositories. Table 1 provides a list of the repositories and the URL for data citation. Each of the repositories follows a regular structure. Specifically, the DICOM files are in the “In” folder, with the validated reference NIfTI format images and BIDS text files in the “Ref” folder. Also in the root folder is the “README.md” text file that describes the rationale for the repository, with each repository showcasing unique DICOM properties. The “LICENSE” file details the permissive license used for distribution. All repositories also include the shell script “batch.sh” that will invoke the user’s installed instance of dcm2niix to generate a new set of images in a folder named “Out” and to report any differences between these and the files provided in the “Ref” folder.

Each repository listed in Table 1 is structured as a standalone dataset with the following top-level folders and files, as shown

- In/: Contains the original DICOM files in.dcm format. Files are organized by series and maintain the original filenames as exported from the scanner or PACS.
  - Ref/: Contains validated reference files in BIDS-compliant format:.nii: NIfTI-formatted imaging data.json: Metadata sidecars with key acquisition parameters (e.g., RepetitionTime, EchoTime, PhaseEncodingDirection, etc.).bvec/.bval: FSL format gradient directions and magnitude for diffusion images.
- README.md: Describes the goal of the repository, key characteristics of the dataset, and instructions for running the validation.
- LICENSE: Declares the BSD 2-Clause license, with a dual-license option under CC BY 4.0 for image reuse.
- batch.sh: A shell script that calls the user’s local dcm2niix installation to convert In/ to Out/, and compares the results against Ref/.

Some repositories also include domain-specific resources:

*.xlsx: Spreadsheet with parameter sweeps or timing calculations (e.g., dcm_qa).
- slicetime.cpp: Minimal C source for slice timing validation (dcm_qa_ge).
*.bval, *.bvec: Diffusion validation code and files (e.g. dcm_qa_dwi).

Folder and file names are consistent across repositories to facilitate automation. Users can expect the same script (batch.sh) and README format in each repository, regardless of modality or manufacturer. The primary variable across repositories is the DICOM content: different manufacturers, modalities, or edge cases (as described in the Comments column of Table 1).

The goal of these repositories is to demonstrate DICOM implementations, and therefore there was an explicit emphasis on low resolution images with a small number of observations. Many of the repositories include phantoms (water bottles). Some modalities (in particular, diffusion and arterial spin labeling) benefit from images of the human brain to allow proper validation. In these cases, data was acquired from the co-authors with the explicit knowledge that these would be shared on public repositories. These images also include generalized demographic details required to estimate specific absorption rate (SAR) such as age, height and weight. The Institutional Review Board (IRB) at the University of South Carolina determined that IRB review was not required for this project, as it does not meet the criteria for “research” as defined under 45 CFR 46.102(l), given that the data from each repository pertains to a single individual and are not generalizable to broader populations. This determination aligns with the intent of the project, which is to provide high-quality validation resources for the neuroimaging community rather than to draw inferences about human health or behavior.

This simple structure allows developers to ensure consistent results across different versions of dcm2niix, including builds compiled with different toolchains, targeting various architectures, or linked against different libraries, all of which can introduce variability in output^21,22^. While these repositories were originally developed to support automated regression testing of dcm2niix^15^, portions have since been adopted to assist development and validation in other tools, including nibabel^23^, divest for r^24^, mriconvert^15^, SPM^15^, FreeSurfer’s mri_convert^25^, orthanc-neuro^26^, and dicm2nii^15^.

Several of the repositories contain additional files that help developers extract the correct results. For example, ‘dcm_qa’ provides an Excel format spreadsheet that demonstrates how in-plane acceleration factor, partial Fourier and other parameters were varied, providing the formulas to infer total readout time. Likewise, the repository ‘dcm_qa_ge’ provides a minimal C program (slicetime.cpp) validated by General Electric (GE) engineers for deriving parameters. Another example is ‘dcm_qa_sag’, which includes Python scripts to generate validation tensor files and bitmap images used to confirm the correct definition of the diffusion gradient directions. In all these cases, these supplemental files are described in the “README.md” file of the repository.

Most of the repositories in Table 1 focus on the Magnetic Resonance (MR) modality, reflecting its versatility in generating diverse contrasts and its popularity among researchers due to the absence of ionizing radiation exposure for participants. The repository ‘dcm_qa_ct’ provides examples of computed tomography (CT), highlighting unique features of this modality, such as gantry tilt (leading to shear in 3D volumes) and variable inter-slice distances.

As shown in Table 1, each validation repository is modular, following the same structure. Typically, each repository is designed to showcase a specific edge case, as indicated by the ‘Comments’ section in Table 1, as well as the individual README.md files within each repository. The README file provides the rationale for each repository. We envision the number of repositories growing to document the evolving development of DICOM usage. Zenodo provides a master repository (‘dcm_validate’) that includes all sub-modules. This provides a single starting location for all of the modules.

In general, most validation repositories provide DICOM images acquired directly from the scanner without modification. However, there are exceptions where images were exported through a local Picture Archiving and Communication System (PACS), and these images can be identified by inspecting the Implementation Version Name (0002,0013) tag. No uniform de-identification tool or configuration was applied across datasets; rather, each dataset reflects the local practices at the institution where the data were acquired. We emphasize that private attributes critical for acquisition metadata were retained.

## Technical Validation

Each dataset includes reference BIDS format text files that have been meticulously checked to ensure accurate correspondence with the DICOM information. As these have been made publicly available, users have been able to identify limitations and extend the content of the JSON files as the BIDS specification gets extended and our understanding of the manufacturer’s own interpretation of the DICOM standard and their use of proprietary DICOM tags improves. Any individual can use a GitHub issue to make a suggestion for how these repositories can be enhanced (with our dcm2niix repository already listing 931 closed issues that describe enhancements, feature requests, and limitations). Therefore, these repositories provide a method for the community to work collaboratively to ensure robust data conversion.

Although every effort has been made to ensure accuracy, certain limitations are inherent in interpreting vendor-specific attributes without formal documentation. In the absence of public manufacturer-issued conformance statements, extracted metadata were manually inspected and, when possible, verified with input from manufacturer engineers. By remaining open-source, these datasets invite community-driven validation and represent the most complete publicly available harmonization effort.

**Fig. 1:**
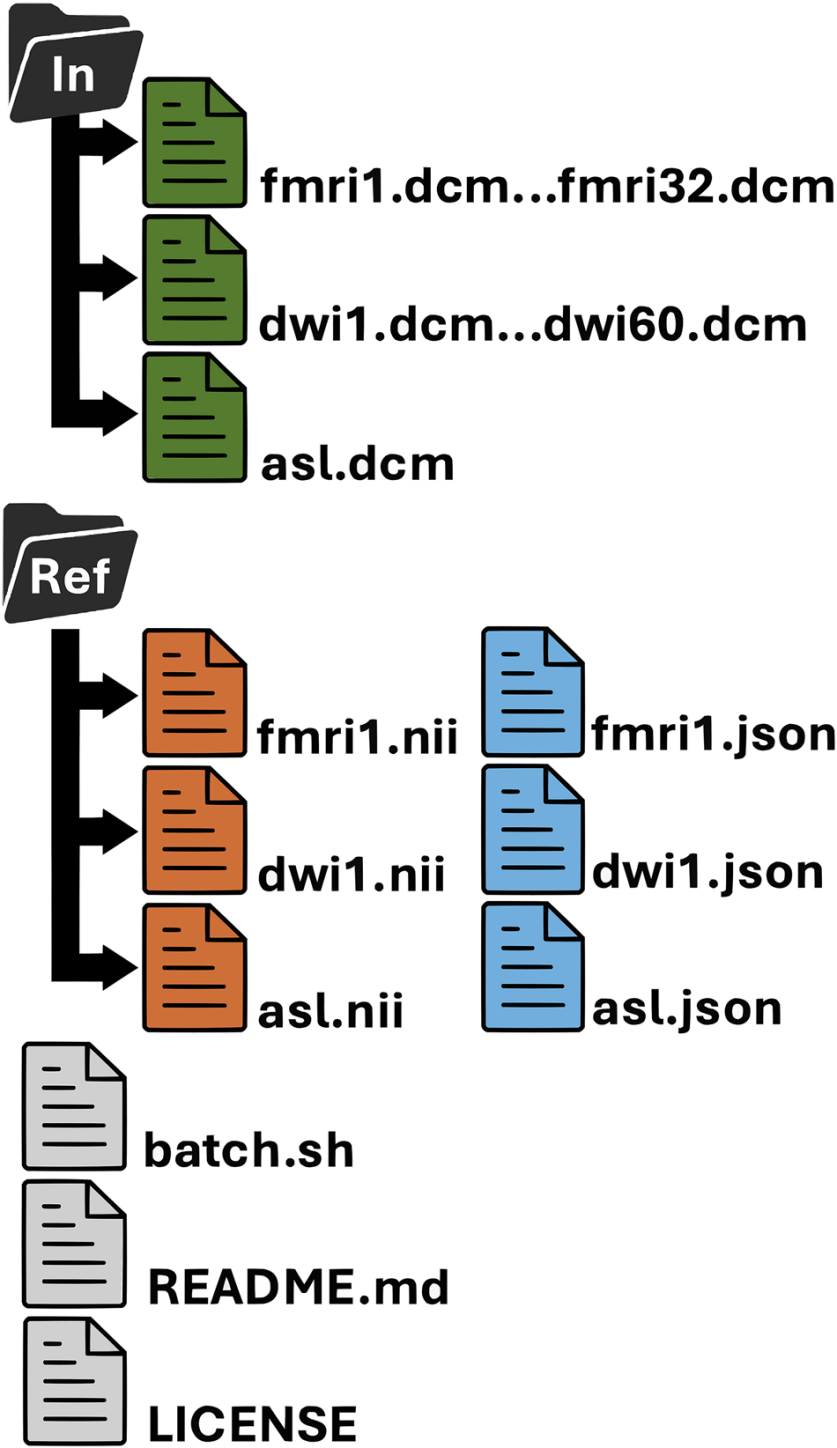
Structure of a Validation Repository. Each repository contains two folders: the “In” folder holds the input files in DICOM format, while the “Ref” folder contains the reference conversion of these DICOM files to BIDS format, with each series producing a NIfTI image file (.nii) and an accompanying JSON metadata file (.json). The figure illustrates three types of 4D time-series inputs: functional imaging (fMRI), stored as mosaics with one DICOM file per 3D volume (32 volumes); diffusion imaging (DWI), saved in classic DICOM format with one file per 2D slice (60 slices); and arterial spin labeling imaging (ASL), saved in enhanced DICOM format, where all slices and volumes are stored in a single file. In all cases, the reference NIfTI files are stored as 4D data. The repository also includes a shell script (batch.sh) that uses dcm2niix to convert the DICOM data from the “In” folder into a BIDS dataset in a new “Out” folder and verifies that the output matches the files in the “Ref” folder. Additionally, a README.md file describes the unique properties of the repository, and a LICENSE file specifies the permissions for sharing the dataset.

To ensure the validity and quality of our own derived datasets, we initially used the provided batch.sh script to generate a reference conversion using dcm2niix, with outputs saved in the Ref folder. The resulting BIDS datasets were then evaluated using the bids-validator to confirm compliance. We manually inspected all fields to ensure that required metadata were either correctly populated or documented as unavailable in the source DICOMs. For each new release of dcm2niix, these repositories are revalidated to ensure identical results; any discrepancies are reviewed manually to determine whether they reflect an unintended change or a meaningful improvement. Sharing these datasets publicly has allowed the broader neuroimaging community—including developers of related tools—to provide feedback and help verify that the curated outputs are both accurate and comprehensive Fig. 1.

## Usage Notes

The validation repositories with DICOM files and their corresponding validated BIDS/NIfTI reference files are publicly available on Zenodo^16^. Once downloaded, each module includes a script “batch.sh” BASH command line script can be executed. This will use the version of dcm2niix in the user’s path to convert all of the DICOM files in the “In” folder to a new folder “Out” and then test that all of the files in the “Out” folder match the reference files in the “Ref” folder. Binary NIfTI images are tested for identical matches, while text-based JSON files are compared key by key, with any discrepancies reported.

To help users navigate the growing collection of validation datasets, we include a Python script (catalog_datasets.py) that catalogs series-level DICOM metadata across all submodules. This script is designed to run in two stages: the first pass scans all available BIDS JSON files (typically one file per DICOM series) and generates a catalog_fields.txt file listing all encountered fields. Users can then edit this file to select a subset of relevant fields (e.g., Manufacturer, PatientAge, EchoTime). A second run of the script uses the customized field list to generate a comma-separated values (CSV) table summarizing the selected metadata across all datasets. This utility facilitates dataset discovery and supports tool developers in identifying representative series for specific testing scenarios.For example, at the time of writing, the field “Manufacturer” appeared in 427 JSON files: Siemens (210), GE (90), Philips (70), Canon (45), Toshiba (5), and UIH (5). Similarly, the “MagneticFieldStrength” field identified 12 series acquired at 1.5 T, 391 at 3 T, and 14 at 7 T, with the remainder corresponding to CT acquisitions. This cataloging script provides a flexible way to search the entire family of repositories, enabling users to identify datasets with specific acquisition properties or scanner configurations without downloading each repository individually.

## Data Availability

All repositories are publicly available with URLs for each dataset listed in Table 1. Each repository is available using a permissive open source license with details described in each repository’s “LICENSE” file. Users can make suggestions directly from any of the repository web pages by generating an “Issue”.
